# Second victim: a concept analysis based on Rodgers’ evolutionary model

**DOI:** 10.15649/cuidarte.5000

**Published:** 2026-06-26

**Authors:** Matheus Tavares França da Silva, Caroliny Cristine dos Santos Mendes, Rebeca Furtado Fernandes, Sherida Karanini Paz de Oliveira, Rhanna Emanuela Fontenele Lima de Carvalho

**Affiliations:** 1 Master's student, Graduate Program in Clinical Nursing and Health Care, Universidade Estadual do Ceará, Fortaleza, Brazil. Email: matheus.tavares@aluno.uece.br Universidade Estadual do Ceará Fortaleza Brazil matheus.tavares@aluno.uece.br; 2 Registered Nurse, Universidade Estadual do Ceará, Fortaleza, Brazil. E-mail: carolcmendes12@gmail.com Universidade Estadual do Ceará Fortaleza Brazil carolcmendes12@gmail.com; 3 Master's student, Graduate Program in Clinical Care in Nursing and Health, Universidade Estadual do Ceará, Fortaleza, Brazil. E-mail: rebeca.furtado@aluno.uece.br Universidade Estadual do Ceará Fortaleza Brazil rebeca.furtado@aluno.uece.br; 4 PhD, Professor, Universidade Estadual do Ceará, Fortaleza, Brazil. Email: karanini@yahoo.com.br Universidade Estadual do Ceará Fortaleza Brazil karanini@yahoo.com.br; 5 PhD, Professor, Universidade Estadual do Ceará, Fortaleza, Brazil. Email: rhanna.lima@uece.br Universidade Estadual do Ceará Fortaleza Brazil rhanna.lima@uece.br

**Keywords:** Patient Safety, Occupational Health, Concept Formation, Seguridad del Paciente, Salud Ocupacional, Formación de Conceptos, Segurança do Paciente, Saúde Ocupacional, Formação de Conceito

## Abstract

**Introduction:**

Healthcare-related incidents affect both patients and professionals involved. The impact on these professionals, known as “second victims,” is one of the major hidden burdens of harm.

**Objective:**

To analyze the concept of the “second victim” from Rodgers’ evolutionary perspective.

**Materials and Methods:**

This study conducted a concept analysis based on Rodgers’ evolutionary perspective, using a scoping review to identify attributes, antecedents, consequences, surrogate terms, and related concepts associated with the “second victim” concept.

**Results:**

The attributes of the “second victim” concept involve persistent physical, emotional, and psychological consequences. Antecedents include a punitive culture and a lack of institutional and peer support. Consequences include sleep disorders, anger, fear, sadness, shame, intrusive memories, anxiety, depression, isolation, absenteeism, and leaving the profession. Related terms include patient safety and mental health, while surrogate terms include the “second victim phenomenon” and “second victim syndrome”.

**Discussion:**

Punitive environments exacerbate negative impacts, undermine patient safety, promote error concealment, and increase underreporting, thereby hindering organizational learning and the correction of failures.

**Conclusion:**

The “second victim” concept refers to healthcare workers, scholars, and administrative or support staff who are directly or indirectly involved in patient care and who, when involved in an unintentional healthcare-related incident, manifest physical, emotional, and psychological reactions with repercussions across biopsychosocial, ethical, and legal dimensions. Without adequate and timely support, these reactions can become chronic and, eventually, pathological.

## Resumen

**Introducción:**

Los incidentes en la atención sanitaria no solo afectan a los pacientes, sino también a los profesionales involucrados, conocidos como “segundas víctimas”. El impacto sobre estos profesionales se considera una de las principales cargas ocultas del daño.

**Objetivo:**

Analizar el concepto de“segunda víctima” desde la perspectiva evolutiva de Rodgers.

**Materiales y métodos:**

La investigación consiste en un análisis de concepto basado en la visión evolutiva de Rodgers, utilizando una revisión de alcance para identificar atributos, antecedentes, consecuencias, términos sustitutos y conceptos relacionados con el concepto de “segunda víctima”.

**Resultados:**

El concepto de “segunda víctima” incluye repercusiones físicas, emocionales y psicológicas duraderas. Los antecedentes incluyen la cultura punitiva y la falta de apoyo institucional y de pares. Las consecuencias abarcan trastornos del sueño, ira, tristeza, ansiedad, depresión, aislamiento, absentismo y abandono profesional. Los términos relacionados son seguridad del paciente y salud mental, mientras que los sustitutos incluyen “fenómeno” y “síndrome” de la segunda víctima.

**Discusión:**

Los entornos punitivos agravan los impactos negativos, desmotivan la seguridad del paciente, promueven que se oculte errores y que no se reporten, dificultando el aprendizaje organizacional y la corrección de errores.

**Conclusiones:**

El concepto de“segunda víctima”se refiere a los trabajadores de la salud, académicos y personal administrativo o de apoyo, involucrados directa o indirectamente en la atención al paciente, quienes, al verse involucrados en un incidente involuntario relacionado con la atención en salud, manifiestan experimentar reacciones físicas, emocionales y psicológicas, con repercusiones en las dimensiones biopsicosocial, ética y jurídica. Sin un apoyo adecuado y oportuno, estas reacciones pueden volverse crónicas y, con el tiempo, patológicas.

## Resumo

**Introdução:**

Incidentes na assistência à saúde afetam tanto os pacientes quanto os profissionais envolvidos. O impacto sobre esses profissionais, conhecidos como "segundas vítimas", é um dos principais fardos ocultos do dano.

**Objetivo:**

Analisar o conceito de “segunda vítima” sob a perspectiva evolucionária de Rodgers.

**Materiais e métodos:**

A pesquisa realiza uma análise de conceito fundamentada na visão evolucionária de Rodgers, utilizando uma revisão de escopo para identificar atributos, antecedentes, consequentes, termos substitutos e conceitos relacionados ao conceito de “segunda vítima”.

**Resultados:**

Os atributos do conceito de “segunda vítima” envolvem repercussões físicas, emocionais e psicológicas duradouras. Os antecedentes incluem cultura punitiva e falta de apoio institucional e de pares. Os consequentes, destacam distúrbios do sono, raiva, medo, tristeza, vergonha, memórias intrusivas, ansiedade, depressão, isolamento, absenteísmo e abandono da profissão. Os termos relacionados abrangem segurança do paciente e saúde mental, enquanto termos substitutos incluem “fenômeno” e “síndrome” da segunda vítima.

**Discussão:**

Ambientes punitivos agravam os impactos negativos, desestimulam a segurança do paciente, promovem a ocultação de erros e aumentam as subnotificações, dificultando o aprendizado organizacional e a correção de falhas.

**Conclusões:**

O conceito de “segunda vítima” refere-se aos trabalhadores de saúde, acadêmicos e colaboradores administrativos ou de apoio, envolvidos direta ou indiretamente no cuidado ao paciente, que ao implicarem-se em um incidente relacionado à assistência à saúde de natureza não intencional, manifestam reações físicas, emocionais e psicológicas, com repercussões nas dimensões biopsicossociais, éticas e legais. As reações sem o suporte adequado e oportuno, podem se tornar crônicas e, eventualmente, patológicas.

## Introduction

Unsafe care, a widely recognized serious public health problem, affects millions of patients worldwide. This phenomenon compromises the reputation of health systems, trust in healthcare services, the well-being of professionals, and public perceptions of resource allocation in the sector. Such incidents can result in death, disability, and suffering for patients and their families, as well as generate economic costs^1^.

Incidents not only directly affect patients but also have consequences for the healthcare professionals involved. The collateral impact on these professionals is considered one of the major hidden burdens associated with adverse events. Affected professionals are described as“second victims,” experiencing detrimental effects on their physical and mental health, as well as an increased risk of new incidents in unsafe care settings^2^.

In this sense, the analysis of the consequences of healthcare-related incidents has expanded to encompass not only the effects on patients and their support networks but also the repercussions for health professionals. The term “second victim” was introduced by Albert Wu in 2000, representing a conceptual milestone in broadening the discussions on the consequences of errors from the perspective of healthcare professionals^3^.

However, at the time of its formulation, a culture firmly centered on the physician prevailed, and patient safety research was still in its early stages, with little attention to systemic and interprofessional dynamics. Thus, the author largely restricted the concept to physicians, making only brief references to other professionals and recognizing, in a limited way, the impacts of errors on other groups of workers^3^.

The concept of the second victim, although introduced more than two decades ago, still lacks a consolidated definition. Its boundaries remain unclear, particularly regarding the identification of the individuals involved and the contextual and relational factors that shape the experience of this phenomenon. The theoretical contributions accumulated since then have been limited, often imprecise, and divergent with respect to who is truly affected, who needs support, what psycho-emotional consequences are involved, as well as their prevalence and duration^4^,^5^.

In the literature, only one evidence- and consensus- based definition of the “second victim” concept has been proposed. However, that research did not employ a concept analysis method nor draw on a theoretical framework for its development, resulting in the absence of identified contextual bases, surrogate terms, and related concepts^5^.

The definition has weaknesses in that it restricts the “second victim” experience to formally trained health professionals, overlooking practicum students and administrative or logistical support staff who, although indirectly involved in clinical care, may also be affected. In addition, the definition does not encompass the specific manifestations of the condition, including physical, emotional, psychological, or social symptoms.

The scenario highlights the need for research that proposes new descriptions or definitions and seeks to build a conceptual consensus. In this sense, recognizing that the current fragility of the concept hinders its reliable implementation, this study aims to structure and operationalize the second victim concept, making it more clearly defined and applicable in practice.

Thus, the study is distinguished by its methodologically rigorous concept analysis based on Rodgers’ evolutionary model. The evolutionary method of concept analysis considers contextual factors that influence the concept of a given term. It is a cyclical process guided by three elements: significance, use, and application. The meaning of a concept depends on its use and application^6^.

Accordingly, this research aims to analyze the concept of “second victim” from Rodgers’ evolutionary perspective. The following research questions guided the study: How is the concept of“second victim” used in the scientific literature? What are the contextual bases, surrogate terms, and related concepts associated with the “second victim” concept according to Rodgers’ evolutionary perspective?

In this sense, the results obtained are expected to promote reflection and foster debate on the subject, with the aim of developing a more consistent conceptualization of the “second victim.” Recognizing this concept may help those involved cope with the incident, thereby validating their thoughts and emotions.

The selection of this concept is justified by its complex construct and the need for a better understanding of how this condition manifests in individuals (its attributes), what underlies its emergence (antecedents), what repercussions (consequences) it entails for professionals in healthcare settings, and how the construct relates to other areas (related terms), particularly in the context of patient safety.

## Materials and Methods

This study is a concept analysis based on Rodgers’ evolutionary approach6 applied to the “second victim” concept. This methodology was chosen because the concept has evolved over time and has been shaped by multiple factors.

The method was implemented following the six steps proposed by Rodgers: identification of the concept of interest; selection of the setting for data collection; data collection to identify attributes and context of the concept; evaluation of the concept’s characteristics; development of a conceptual model; and identification of hypotheses and implications^6^.

In the first step of the method, the selected concept was the “second victim.” The data collection setting was based on a scope review conducted following the Joanna Briggs Institute (JBI) guidelines^7^ and the PRISMA-ScR checklist^8^, given their suitability for mapping the literature, identifying gaps, and clarifying theoretical concepts.

The scoping review was registered in the Open Science Framework^9^ and is available in full. The study protocol can be accessed to provide a more detailed understanding of the methodological steps presented below.

The research question was formulated using the PCC framework (P: Population/C: Concept/C: Context), with healthcare workers as the population, the “second victim” as the concept, and patient safety as the context. The guiding question focused on the attributes, antecedents, and consequences of the “second victim” concept in the context of patient safety, as follows: “What are the attributes, antecedents, and consequences of the second victim concept among healthcare workers in the context of patient safety?”

The scoping review included publications addressing the attributes, antecedents, and consequences of the concept, with no language restrictions and a time frame starting in 2000, when the term was first documented. Primary empirical studies, quantitative and qualitative, were included, while incomplete, duplicate, preprint, or non-open-access articles were excluded. The identification of contextual bases (antecedents and consequences), surrogate terms, and related concepts was guided by the following questions, as presented in Table 1.

**Table 1:** Items and guiding questions used in Rodgers’ evolutionary concept analysis method

Item analyzed	Guiding question
Antecedents	What events contributed to the emergence of the term “second victim”?
Consequences	What are the consequences associated with the use of the term “second victim”?
Surrogate terms	What words or expressions are used as substitutes for the term “second victim”?
Related concepts	What philosophical assumptions shape the meaning of the term “second victim”?
Attributes/Concept	How do authors define the second victim? What are the main characteristics presented by the authors associated with the meaning of the term “second victim”?

The search strategy used the terms “Health Personnel,” “Medical Errors,” and “Patient Safety,” selected from Medical Subject Headings (MeSH) and Health Sciences Descriptors (DeCS). Equivalent terms in English, including synonyms in both singular and plural forms, were considered. The Boolean operator “and” was used to combine search terms as appropriate. The search strategy was adapted to each database's specific syntax and controlled vocabulary.

The searches were conducted in October 2024 across the following databases: Scientific Electronic Library Online (SciELO), Scopus, Medical Literature Analysis and Retrieval System Online (MEDLINE) via PubMed, Web of Science, ScienceDirect, and the Virtual Health Library (VHL), which includes the Latin American and Caribbean Health Sciences Literature (LILACS), the Spanish Bibliographic Index of Health Sciences (IBECS), and Nursing Database (BDENF, in Portuguese). Grey literature was searched using Google Scholar and the Brazilian Digital Library of Theses and Dissertations (BDTD, in Portuguese).

The results retrieved from the databases were exported to the Rayyan® reference manager tool to remove duplicates and to enable independent selection and screening of studies by two reviewers, with any disagreements resolved through discussion until consensus was reached.

The first phase involved screening the titles and abstracts of the retrieved studies. Studies that met the inclusion criteria were assessed in the second phase through full-text review. The reviewers read the full texts and applied the eligibility criteria to select the studies.

The data extraction form included fields for title, authors, country, year, study design, and population. The extracted data, as well as the PRISMA flow diagram detailing the process leading to the final sample that informed the concept analysis, are available in Mendeley Data for open access and consultation^10^ and in the full report of the scoping review^9^.

The data extracted from the selected articles enabled the identification of the concept’s attributes, including its definition, antecedents, consequences, sociocultural and temporal variations, surrogate terms, and related concepts. The review of the studies facilitated an in-depth understanding of these aspects, enabling the extraction of data relevant to the analysis.

The third step, data analysis, was conducted after collecting and organizing all the information. The elements constituting the concept were examined based on the literature. The frequency of terms and their meanings was evaluated, highlighting similarities and discrepancies to identify the most commonly used terms for describing the concept. Subsequently, a practical example was developed to illustrate the concept. Finally, guidelines for future research were presented, emphasizing the concept's applicability and evolution among healthcare workers in the context of patient safety.

## Results

The final sample of the review comprised 40 articles and one dissertation, published across 13 countries, with the highest number from the United States (16), followed by Brazil (6) and Spain (5). Other countries, such as Korea, Belgium, and Austria, contributed two publications each, while Germany, Saudi Arabia, Italy, Denmark, Canada, Ireland, China, and Colombia each contributed one article. Most studies were published in English (35), while five were in Portuguese and one was in Korean.

Regarding the methods employed, 17 studies used a cross-sectional design, with a predominance of qualitative approaches (8), which enabled in-depth analysis of the perceptions and challenges faced by second victims. Other studies employed mixed-methods (3), observational (1), descriptive (1), and exploratory (1) designs. In addition, editorial articles (5), commentaries (2), and informative papers (1) played a relevant role in the development and discussion of the concept within the scientific field. There was also an increase in publications over the past six years (2019-2024), with 25 studies published during this period, indicating a growing interest in the topic over time.

The study samples included different professional groups identified as potential second victims. The most frequently reported were nurses, nursing technicians, and nursing assistants (30), followed by physicians (18), practicum students and healthcare residents (12), pharmacists (3), physical therapists (2), social workers (1), laboratory technicians (1), physiopathology technicians (1), and cleaning staff (1). Several studies reported more than one professional group, underscoring the extent of the phenomenon across different healthcare actors. However, in some studies (4), authors referred to healthcare professionals in general without specifying professional groups.

The included studies sought to understand the experiences of second victims, emphasizing the impacts of adverse events in healthcare. The evolution of the concept has expanded the range of professionals beyond formally trained healthcare workers, underscoring the need to provide adequate support, strengthen patient safety, and adopt a more humanized approach. However, many studies continue to refer to these professionals in general terms, without specifying their professional group.

The remaining data were organized according to the categorization proposed by Rodgers, which includes three main dimensions: antecedents and consequences, which represent the factors associated with the concept and its ramifications; surrogate terms and related concepts, which contribute to its definition; and attributes, which describe its essential characteristics.

**1. Analysis of attributes and context**

**Figure 1:**
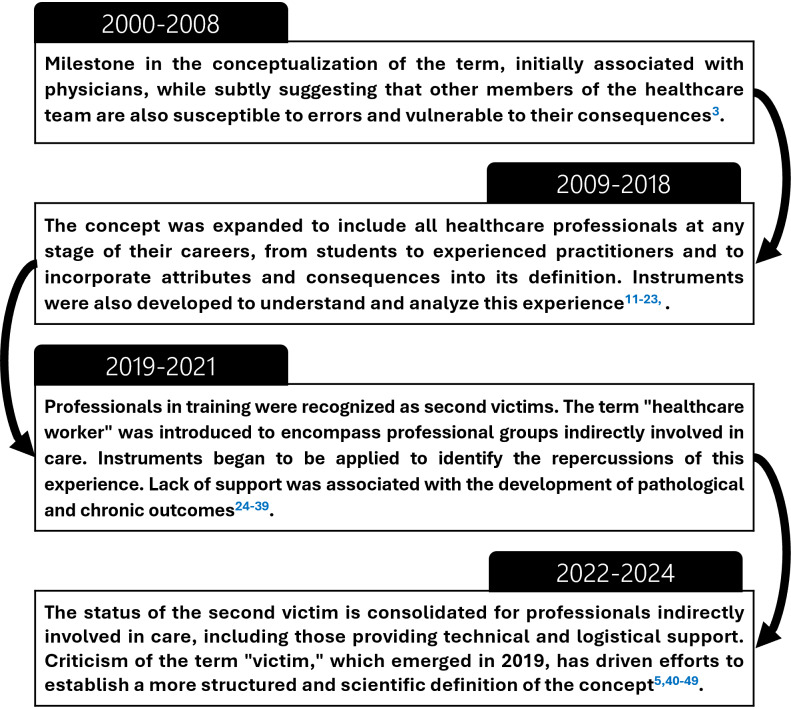
Evolution of the “second victim” concept over time. Fortaleza (CE), Brazil, 2024

The analysis of the studies enabled the identification and chronological organization of definitions attributed to the “second victim” concept. The compilation of definitions illustrates the concept’s maturation, reflecting changes in its conceptual development and highlighting variations in its interpretation, as well as their potential implications for practice. Figure 1.

*See references for Figure 1: ^3^,^11^,^23^,^24^-^39^,^5^,^40^-^49^*

The definition of the “second victim” concept has progressively expanded over time as research has deepened understanding of healthcare professionals’ experiences. The process of scientific inquiry has contributed to refining the concept, reflecting the complexity of the situations encountered.

**2. Analysis of data in relation to the characteristics of the concept Antecedents and attributes of the “second victim” concept**

The concept of“second victim”is preceded and influenced by several factors, such as situations, events, and phenomena. Studies indicate that a weakened patient safety culture, combined with the absence of support mechanisms, facilitates the emergence of this condition among healthcare professionals. The interaction between organizational culture and worker well-being is crucial for understanding how adverse experiences in healthcare affect the health of those involved. Table 2.

**Table 2:** Distribution of antecedents and attributes of the second victim concept, Fortaleza, Brazil, 2024

Antecedents	Number of citations % (n)
Lack of institutional support mechanisms^3^,^11^-^13^,^15^,^17^-^20^,^22^,^26^,^29^,^31^,^33^,^37^,^41^,^43^,^44^	43.90 (18)
Punitive culture^3^,^12^,^18^,^20^-^22^,^26^,^31^,^32^,^34^,^37^,^38^,^40^,^47^,^48^	35.50 (15)
Reluctance to use support services^12^,^13^,^15^,^16^,^20^,^22^,^31^,^37^,^38^,^44^,^47^	26.80 (11)
Lack of peer support^3^,^15^,^20^,^21^,^22^,^26^,^29^,^37^,^43^	21.90 (9)
Lack of transparency in case management^11^,^15^,^17^,^20^,^21^,^26^,^33^,^37^	19.50 (8)
Failure to disclose^12^,^13^,^15^,^20^,^21^,^26^,^33^	17.00 (7)
Culture of perfection^3^,^11^,^14^,^22^,^35^,^37^,^41^	17.00 (7)
Insufficient protocols^12^,^26^,^40^	7.30 (3)
Inadequate crisis management^12^,^30^	4.80 (2)
Attributes	
Persistent/long-lasting^11^-^15^,^18^-^20^,^26^,^28^-^31^,^33^-^36^,^38^,^41^,^45^,^47^	51.90 (21)
Emotional impact^3^,^11^-^16^,^18^-^22^,^26^,^28^	35.50 (15)
Psychological impact^3^,^12^,^15^,^17^-^21^,^23^,^26^,^29^,^31^,^45^,^46^	34.10 (14)
Physical impact^11^,^12^,^14^,^15^,^17^-^21^,^26^,^31^,^35^	29.20 (12)
Ethical, legal, and juridical impact^11^,^14^-^16^,^19^-^22^,^29^,^32^,^43^	26.80 (11)
Moral impact^12^-^17^,^20^,^31^,^35^,^45^	24.30 (10)
Social impact^3^,^11^,^12^,^14^,^15^,^18^,^20^,^23^,^31^,^47^	24.30 (10)

The identified attributes indicate that the second victim condition affects the biopsychosocial aspects of the professionals involved, resulting in persistent consequences. In addition, they highlight the significance of the emotional, physical, and social consequences these professionals face, underscoring the need for adequate support.

**Consequences of the “second victim” concept**

The consequences related to the second victim concept highlight its impact on workers’ health and on personal and professional performance. The results indicate that these effects may compromise individuals’ physical and emotional integrity. The analysis of these repercussions highlights the importance of a holistic approach that accounts for the multiple factors influencing the lives and work of these professionals. Table 3.

**Related concepts and surrogate terms of the “second victim” concept**

The concepts related to the “second victim” are intrinsically linked to patient safety and its impacts on workers’ health, with particular emphasis on the psychological dimension. However, the surrogate terms identified are more related to the manifestations arising from this experience, in terms of the chronicity and progression toward pathological responses, than to the individuals themselves. Table 4.

**The “second victim” concept**

The identification of the fundamental elements of the concept enabled a clearer definition of the concept and its scope of application, which are directly related to the characteristics of healthcare professionals’ lived experiences.

In this regard, the conceptual definition of the second victim proposed in this study refers to healthcare workers, scholars, and administrative or support staff who are directly or indirectly involved in patient care and who, when involved in an unintentional healthcare-related incident, experience physical, emotional, and psychological reactions with repercussions across biological, social, ethical, and legal dimensions. Without adequate and timely support, these reactions may become chronic and, eventually, pathological.

**Table 3:** Distribution of consequences related to the second victim, Fortaleza, Brazil, 2024

Consequences	Number of Citations % (n)
Physical Consequences	
Sleep disorders^11^,^12^,^14^,^15^,^17^-^21^,^23^,^28^,^29^,^31^-^33^,^35^,^36^,^38^,^42^,^43^,^45^,^48^,^49^	63.40 (23)
Fatigue^11^,^16^,^17^,^19^,^20^,^21^,^26^,^32^,^35^,^42^,^48^	26.90 (11)
Changes in vital signs^11^,^18^,^19^,^42^,^48^,^49^*	14.60 (6)
Muscle tonus^19^,^26^,^32^,^48^,^49^	12.10 (5)
Loss of appetite^20^,^32^,^35^,^49^	9.70 (4)
Headache^19^,^45^,^46^,^48^	9.70 (4)
Nausea^31^,^32^,^42^,^48^	9.70 (4)
Pain^11^,^31^,^32^	7.30 (3)
Vomiting^20^,^48^	7.80 (2)
Abdominal pain^19^,^20^	7.80 (2)
Weight loss^20^	2.40 (1)
Emotional Consequences	
Guilt^3^,^11^,^12^,^14^-^17^,^20^-^23^,^26^,^28^-^30^,^32^,^33^-^38^,^42^,^43^,^45^,^47^,^48^	65.80 (27)
Anger^3^,^11^,^12^,^14^,^15^,^18^-^23^,^26^,^28^,^32^,^33^,^35^,^38^,^43^,^45^,^46^,^49^	51.20 (21)
Shame^13^-^16^,^20^-^23^,^26^,^28^,^29^,^33^-^37^,^47^,^48^	43.90 (18)
Fear^12^,^15^,^16^,^18^,^23^,^29^,^31^,^32^,^35^-^37^,^41^,^47^,^48^	34.10 (14)
Sadness^11^,^16^,^19^,^20^,^22^,^26^,^28^,^36^,^43^,^48^,^49^	26.80 (11)
Reduced job satisfaction^11^,^15^,^18^-^21^,^23^,^26^,^32^,^33^,^49^	26.80 (11)
Remorse^11^,^15^,^16^,^19^,^26^,^31^,^37^,^49^	19.50 (8)
Concern^14^-^16^,^18^,^26^,^31^,^49^	17.00 (7)
Helplessness^12^,^14^,^15^,^20^,^26^	12.10 (5)
Disappointment^20^	2.40 (1)
Grief^26^	2.40 (1)
Psychological Consequences	
Loss of self-confidence^3^,^11^-^20^,^23^,^26^,^28^-^30^,^32^-^34^,^36^,^38^,^40^,^42^,^45^-^48^	68.20 (28)
Anxiety^3^,^11^-^20^,^22^,^23^,^26^,^29^,^30^,^33^,^34^,^38^,^43^,^45^,^48^,^49^	58.50 (24)
Repetitive/intrusive memories^3^,^11^-^14^,^17^,^19^,^20^,^23^,^26^,^29^,^31^,^33^-^36^,^41^-^43^,^46^,^47^,^49^	53.60 (22)
Depression^11^-^15^,^19^-^21^,^26^,^29^,^30^,^32^,^37^,^42^,^45^	36.50 (15)
Decreased concentration^11^,^12^,^14^,^15^,^17^,^19^,^20^,^26^,^34^,^38^,^43^,^45^	31.70 (13)
Post-Traumatic Stress Disorder^11^,^12^,^15^,^17^,^20^,^21^,^23^,^29^,^32^,^33^,^35^	24.30 (10)
Frustration^11^,^19^,^20^,^23^,^26^,^34^,^38^,^49^	19.50 (8)
Suicide^14^,^27^,^29^,^38^,^45^,^46^	14.60 (6)
Hypervigilance^3^,^23^,^32^,^36^,^47^	12.10 (5)
Burnout^21^,^31^,^37^,^42^	9.70 (4)
Social Consequences	
Leaving the profession^11^,^12^,^19^,^20^,^23^,^28^-^33^,^35^,^41^,^43^,^45^,^46^	39.00 (16)
Isolation^3^,^11^,^12^,^14^,^15^,^18^-^20^,^29^,^31^,^32^,^41^,^45^,^46^,^48^	29.20 (12)
Absenteeism^17^,^20^,^23^,^26^,^28^,^31^,^33^,^35^	26.80 (11)
Alcohol and drug use and abuse^3^,^31^,^37^,^45^	9.70 (4)
Strain in relationships (children or partners)^20^,^23^,^43^	7.30 (3)
Moral Consequences	
Damage to reputation and professional prestige^11^,^12^,^14^,^15^,^17^-^20^,^26^,^31^,^37^,^38^,^41^,^43^,^45^,^47^,^49^	41.40 (17)
Humiliation^18^	2.40 (1)
Legal Consequences	
Litigation^11^,^14^-^16^,^19^-^22^,^29^,^32^,^43^	26.80 (11)

*Increased heart rate, respiratory rate, and blood pressure.

**Table 4:** Distribution of related concepts and surrogate terms for the “second victim” concept, Fortaleza, Brazil, 2024

Related concepts	Number of citations % (n)
Patient safety^3^,^11^-^14^,^17^,^20^-^24^,^27^,^29^-^38^,^40^,^42^,^43^	63.40 (26)
Worker’s health^3^,^11^,^15^,^23^,^26^-^28^,^30^,^31^	17.00 (7)
Mental health^12^,^17^,^23^,^28^,^38^,^44^	17.00 (7)
Surrogate terms	
Second victim phenomenon^11^,^14^,^17^,^20^,^21^,^24^,^26^,^32^,^33^,^35^,^38^,^44^,^46^	31.70 (13)
Second victim syndrome^13^,^18^,^22^,^24^,^30^,^31^,^46^	17.00 (7)

**Figure 2:**
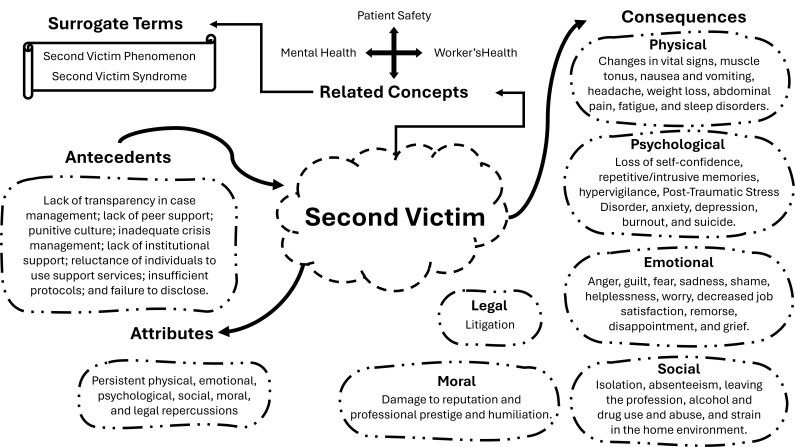
Conceptual structure of the second victim concept and its relationships among

Thus, the concept represents a multifaceted phenomenon intrinsically related to the historical background and culture of healthcare services. Its characteristics highlight relevant concerns, especially regarding the mental health and well-being of healthcare professionals in the context of patient safety. To facilitate understanding of the interrelationships within this construct, Figure 2 is a graphical representation of the concept’s nature and interconnections.

**3. Identification of a model case for the concept**

In this analysis, a real case reported in the literature was used, with the appropriate adaptations to protect the identity of those involved. The selected practical example illustrates the “second victim” concept, highlighting its key attributes and consequences. This case aims to highlight the concept's characteristics, provide an accurate understanding, and facilitate its application.

“An experienced pediatric intensive care nurse, recognized for her dedication, made a medication error by administering an excessive dose of calcium chloride to an infant with severe heart problems.

The error occurred due to a momentary distraction during medication preparation. Although she promptly recognized the error, reported the incident, and intervened to stabilize the patient, she was overwhelmed by intense feelings of guilt and remorse for having caused harm to the infant, who died five days later. It is believed that the error exacerbated the child's heart condition, contributing to the fatal outcome.

The case received widespread media coverage, prompting the nursing board to initiate an investigation. During the proceedings, the institution placed the nurse on administrative leave without providing adequate emotional or professional support. In the absence of peer support, she began to question her professional competence, feeling deeply frustrated, ashamed, and helpless.

After the investigation was completed, the institution terminated her employment contract, and the nursing board imposed sanctions, including a fine and a four-year probationary period during which her medication administration would be supervised. The public repercussions and professional consequences further aggravated her emotional state, leading to insomnia, anxiety, and severe depression. She began to repeatedly relive the incident in her thoughts and dreams, progressively withdrawing from friends, family, and her religious community.

Despite several attempts to re-enter the workforce, the nurse encountered barriers, which intensified her isolation and despair. Faced with the prospect of no longer being able to practice her profession, persistent sadness and feelings of inadequacy culminated in her decision to take her own life seven months after the incident. The impact of her death was profound, with former patients and their family members attending a memorial service in her honor, where they expressed gratitude for her compassion and dedication throughout her career.”

**4. Hypotheses identification and their implications**

The analysis of the “second victim” concept based on Rodgers’ evolutionary approach offers a broader and more practical understanding of the construct. This proposal enables the integration of the concept into discussions on patient safety and workers’ health.

However, studies on second victims have examined the experiences and perceptions of formally trained healthcare professionals. Future research on the topic should adopt a broader scope. The conceptual framework proposed in this research underscores the need to include other healthcare workers who are indirectly involved in care, share responsibility for patient safety, and may also be affected as “second victims.”

It is also important to emphasize the need for future studies that differentiate between the “second victim phenomenon” and “second victim syndrome.” These terms are often used interchangeably with “second victim,” yet they lack a precise conceptual definition.

## Discussion

**Concept contributions**

The “second victim” concept presented in this study revisits and expands the understanding of this condition by incorporating new dimensions into its definition. The proposal’s distinguishing feature is its comprehensive approach, which includes not only physicians but all healthcare workers, scholars, and administrative or support staff as potentially affected individuals. The definition also encompasses the physical, emotional, psychological, social, ethical, and legal consequences these individuals experience, highlighting the need for timely support to prevent chronic effects and associated diseases.

The inclusion of other healthcare workers, such as nurses, physical therapists, and pharmacists, as well as other non-traditional healthcare roles, such as laboratory technicians, cleaning staff^45^, radiotherapy professionals, and porters^17^, in addition to practicum students^30^,^31^,^35^,^36^,^40^,^49^, represents a step forward in understanding the vulnerability of these groups. However, many of these professionals were generically classified as“others” in the studies, highlighting the need for greater recognition and formal inclusion of these groups in discussions on patient safety and exposure to events that may render them potential second victims.

The recognition that other professional categories, whether in training or indirectly involved in patient care, can also become second victims represents a significant contribution. This finding is a crucial step toward ensuring that educational institutions and hospitals develop more comprehensive support programs, so that no individual, regardless of their role or career status, is left without support in times of crisis.

The incorporation of repercussions and consequences into the definition, in turn, supports the conclusion that the resulting harm also affects healthcare workers. Such recognition is essential for planning practical approaches to support these professionals and promote actions that facilitate their recovery and prevent future adverse effects. A systematic review with meta-analysis revealed that the second victim syndrome affects 58% of professionals over the course of their careers, of whom 60% recover within one month, while 20% take more than a year to recover or do not recover^50^.

Thus, including appropriate and timely support as an essential component of managing these experiences and recognizing its critical role in preventing progression toward chronic and pathological responses serves as a warning and holds health institutions accountable for developing support strategies for professionals to prevent these unfavorable outcomes.

Support mechanisms must be accessible, structured, and widely disseminated to ensure positive outcomes and encourage constructive approaches to addressing second victims’ experiences^41^. In this context, organizational culture directly influences the suffering of these professionals and may either mitigate or intensify it. Punitive environments exacerbate adverse effects, undermine a culture of patient safety, promote the concealment of errors, and increase underreporting, thereby hindering organizational learning and the correction of failures^40^.

**Contextual basis**

The concept’s antecedents highlight how a weakened patient safety culture contributes to the negative impacts on second victims. Punitive organizational cultures exacerbate emotional symptoms. These cultures, characterized by blame and fear-based management, compromise professional performance and hinder efforts to prevent new adverse events^51^. Therefore, promoting a patient safety culture based on transparency, without blaming or punishment, and continuous learning is essential for advancing organizational improvements^48^.

The attributes identified as essential to the concept reflect the prolonged and multidimensional nature of the experience, encompassing physical, emotional, psychological, social, moral, and legal repercussions, and highlighting the depth of its impact. The manifestations are particular and affect social, cultural, emotional, spiritual, and physical areas^52^.

Recurrent symptoms, such as hypervigilance, flashbacks, and feelings of shame, may persist for months or even years, especially in the absence of adequate institutional support^5^. When left untreated, these symptoms may progress to severe conditions, such as post-traumatic stress disorder, anxiety, depression, and suicidal ideation^53^. Although some individuals recover in the short term, others experience manifestations that may last a lifetime^17^, underscoring the need for effective interventions to prevent chronicity and worsening outcomes.

The discussion of duration, chronicity, and progression toward pathological responses in the second victim condition is intertwined with the surrogate terms identified in the analyzed studies. The expressions “second victim phenomenon”^21^,^26^,^33^,^35^,^38^,^45^,^46^,^49^ and “second victim syndrome”^13^,^18^,^30^ are often used interchangeably with “second victim.” However, based on the literature reviewed, these terms are more related to lived experiences than to the individuals themselves. Nevertheless, these expressions reflect different aspects: “phenomenon” refers to the symptomatic manifestations following the incident, whereas“syndrome” is associated with cases in which the professional develops a pathological condition due to a lack of resources to cope with the experience.

The second victim condition is closely related to other important concepts, such as mental health, workers’ health, and patient safety, as extensively discussed in the study. These areas converge in addressing worker well-being, highlighting the importance of preventive and supportive measures in situations of work-related trauma. The relationship between occupational health and patient safety has gained attention, as adequate working conditions directly influence the quality of care and patient safety^1^.

Ensuring service quality requires continuous investment in education, training, and the maintenance of professionals’ health. However, high rates of illness among workers reveal a concerning reality, exacerbated by the absence of institutional policies aimed at balancing worker safety and patient safety. This scenario highlights the importance of implementing integrated actions that ensure this essential combination: safe workers and safe patients^54^.

**Critical analysis of the concept**

The analysis of the manuscripts included in this study indicates that, although the “second victim” concept has become established over time, its use remains controversial. Concerns have been raised abput the appropriateness of using the term “victim” to describe healthcare workers, as it may convey an idea of passivity, imply exemption from responsibility, and minimize the need for ethical and professional accountability.

Critics of the term “second victim” argue that it may conflict with patient safety culture, divert attention from the needs of affected patients and their families, and place excessive emphasis on professionals at the expense of a balanced approach^24^. According to them, the terminology may minimize or undermine patients’ experience by suggesting that“everyone is a victim,” thereby diluting the real pain experienced by patients and their families.

In addition, critics point out that this expression may convey the perception that professionals are more concerned with themselves than with their patients. For this reason, some researchers advocate reevaluating and, if necessary, replacing the term with more appropriate alternatives developed in consultation with patients and professionals^39^.

By contrast, proponents of the term “second victim” argue that it reflects the reality of professionals affected by errors arising from flawed systems, highlighting the need for institutional support and an approach focused on organizational well-being. The term is also seen as raising institutional awareness and promoting a patient safety culture that includes support for workers as an essential component of learning and prevention strategies^25^.

Supporters contend that introducing the term has been beneficial, as it has brought attention to the impact of errors on healthcare professionals without diminishing patients’ experiences. They emphasize that, although patient-centered care is essential, the well-being of professionals is equally crucial to the safety and quality of care. They further argue that the focus should be on preventing new incidents through collaboration among patients, family members, and professionals, moving beyond terminological debates and prioritizing practical support and prevention strategies^27^.

The creator of the term, Albert Wu, acknowledges that, despite criticism, the concept is well established and understood among healthcare professionals, and that changing it could lead to confusion. He argues that the term serves its purpose of drawing attention to the problems professionals face. However, he also admits that there are valid arguments both in favor and against its use. In this regard, he suggests that, at present, it is essential to allow advocates to adopt the terminology with which they feel most comfortable, provided that the goal remains the recognition of the problem and the implementation of appropriate solutions^29^.

The objective of this study was to conduct a concept analysis of the term “second victim” with the aim of establishing a more precise definition of the individuals involved and the repercussions of adverse events on healthcare workers. The study does not seek to reformulate the existing terminology; however, it acknowledges that the term “second victim” may not be the most appropriate for referring to these professionals. Accordingly, there is a need for further studies to propose alternative, equally representative terminologies that preserve the impact and relevance of the current term, while promoting greater precision and acceptance in academic and practical fields.

As a concept analysis, this study has limitations. Although it provides a historical and dynamic contextualization of the concept, it relies on the researchers’ interpretations, which may introduce bias and limit the generalizability of its propositions. Furthermore, the decision to include only open-access articles may have limited the sample analyzed, excluding relevant studies available in restricted-access databases. Nevertheless, the study contributes to the conceptual development and definition of the concept and may inform future research. In this sense, further research is recommended to integrate approaches that strengthen the practical application of the concept.

## Conclusions

The concept analysis based on Rodgers’ evolutionary model provided a more accurate understanding of the “second victim” concept, with relevant implications for patient safety. Accordingly, the “second victim” concept refers to healthcare workers, scholars, and administrative or support staff who are directly or indirectly involved in patient care and who, following an unintentional healthcare-related incident, experience physical, emotional, and psychological reactions with repercussions across the biopsychosocial, ethical, and legal dimensions. Without appropriate and timely support, these reactions may become chronic and, eventually, pathological.

The identified antecedents indicate that weaknesses in patient safety culture and the absence of support mechanisms favor the emergence of the second victim condition among healthcare professionals. The influence of historical and cultural factors contributes to the perpetuation of suffering, reinforcing reluctance to seek help and maintaining an image of perfection in healthcare. In this context, the second victim experience warrants reflection on the physical, emotional, and psychological reactions that characterize it and underscore the need for appropriate support.

The consequences of this condition affect both professionals’ well-being and patient safety, as well as organizational dynamics, compromising the quality of care and workers’ performance. Accordingly, concepts related to the “second victim” are intrinsically linked to patient safety and workers’ health, highlighting the need for effective institutional approaches to prevention and support to minimize its negative effects and promote a safer and more supportive work environment.
